# Supplementary material for: Patterns of Intron Gain and Loss in Fungi
Source: PLoS Biol. 2004 Nov 30;2(12):e422. doi: 10.1371/journal.pbio.0020422 (PMC532390; doi:10.1371/journal.pbio.0020422)
Supplement: Table S1 — Also available at http://genes.mit.edu/NielsenEtAl/. (4.3 MB ZIP). [file pbio.0020422.st001.zip › NielsenEtAl/html/575.html]

AN5656.1.NCU01411.1.MG09502.1.FG10120.1


```
 CLUSTAL W (1.82) Multiple Sequence Alignments - Introns Inserted


Sequence 1: NCU01411.1	575 aa
Sequence 2: MG09502.1	565 aa
Sequence 3: AN5656.1	568 aa
Sequence 4: FG10120.1	559 aa
Alignment Length: 594 aa
Number Identitical Residues: 185 aa
Alignment Score (without introns) 10778


MG09502.1 	MEKSGEKAALDSLGRPSSVGASQPANVEYDTSTPEGSVNEKSRRSSREWAEESSESHQAN
NCU01411.1	---MDERHPQKESPRPVALTNAQ----ETLTGGVEEKKGVKEQVTSSSSDTDGPEVVDSS
FG10120.1 	MKSESEQEKRREDKKEVDNGD------EKTSLGPDEESQRPPTSGSTSSKSEH-HVQQDT
AN5656.1  	---MQEKSVVRTQSPERSY--------EAQDSTPISNYQDDLHSLSSSQNSEHSEPEHAT
          	     *:                    *        .        * .   : ..  . .

MG09502.1 	GEEVG-EGSDAEPQTSATAPRAASRASSARSRALSVVPTSRRRGLFARFAVFVPEVYRPF
NCU01411.1	DHEPGPEQDKPHPGGDSALSQPYRTLSSSRVHTACVVARADRRGLLASFA-FIPEVERPV
FG10120.1 	IEPVMLGEPLGHIDTRPSVKRAQSRASSAQSKTRSVVPRSQRRGLLARFT-LVPEIEYPP
AN5656.1  	--TPGRDAEDQVP--------AKSALDSESEEAMKVVPRLSRRGLFGQIT-LLEEIENPK
          	                     .    .*   .:  **.   ****:. :: :: *:  * 

MG09502.1 	DYSNKTKWTITFIVALAASAAPMGSGILYP~VLTDVSVDLKTSPTIVNLTVAVYALAMAI
NCU01411.1	EYKNKTKWMITTIVALAAAGAPFGSGIFLP1ALELMADDLHTSPTITNLAVALYMLAMSI
FG10120.1 	DYKNSTKWCLTAIVALATAAAPLGSTVVYP1ALPILVDEFNTTETITNLSVALYMISMAI
AN5656.1  	CYPRSKKWFITFVVAVAGSVAPMGSSIFFP~ALSQVADELNATTTVTNLTISLYMLSMSI
          	 * ...** :* :**:* : **:** :. * .*  :  ::::: *:.**::::* ::*:*

MG09502.1 	FPIWW2SSFSETFGRRTVYIVSFGLGVVFSILSAVSSNIAMLIVMRMLGGGASAS~VQAV
NCU01411.1	FPLWW~SSFSETLGRRTIYIASFTLFVVFSVLSAVSVNTAMLVIMRLLAGGASAS0VQAV
FG10120.1 	FPLWW~SSFSEEFGRRTIYLISFSLFVIFSILSAISKNITMLIIFRMCAGGASAS~AHST
AN5656.1  	FPLWW~SSFSERLGRRTIYIASFALFVVFNVLCAVSSSIAMLIVMRLLSGGASAS~VQAV
          	**:** ***** :****:*: ** * *:*.:*.*:* . :**:::*: .****** .::.

MG09502.1 	GAGTIADIWEIRERGKAMGMF0YLGPLMGPLLAPIVGGALAQKWNWRSTMW2RKEPE---
NCU01411.1	GAGTIADIWKPAERGRAMGIF~YLGPLIGPLLSPIIGGALSQGFGWRSTMW~FLAIYGSC
FG10120.1 	GAGSIADMFEVFERGRAMSIF~YLGPLLGPMIAPVVGGALTQEIGWQATMW~FLAIYGLV
AN5656.1  	GAGTIADIWDTRERGRAMGIF~YLGPLCGPLIAPIVGGALAQRWHWRSTMW~FFSAYGGV
          	***:***::.  ***:**.:* ***** **:::*::****:*   *::***      .  

MG09502.1 	-------QLPRTVDRNS------TEDQP-----QLSRATTRQS-----IAKSTKHSVKTV
NCU01411.1	TLILIIFALPETLTKRK------PLPPPPAENNELKRVSTRQS-----VAEHSKRVGAIT
FG10120.1 	VLLMILFFLPETLQRKP------EAVLPTADTQELSRMRTMDS-----AKIKTRNFAKSA
AN5656.1  	VVVLILLGLPETLSGANQKPLMPPADPASTTEEPLSRRASRVSSVQTQVFGTTARWLKLI
          	        **.*:    ...  .    ..:  . *.*  :  *: .:.    : .     

MG09502.1 	KRYVFDPLKVLGTLRFLPIAVTVFIAAVTFGSLFILNISIQSSYSKAPYGFSTLIVGLLY
NCU01411.1	RRFIIEPLEVLLYLRYPPVLVSVYSAAIAFGALFILNISIQTTFHSAPYGYSTIIVGLLY
FG10120.1 	RRFLIDPLGVLLYLRFPPVLITVLLAAIAFGSLYVVNIAIQQKFSREPYNFGQLSIGLMY
AN5656.1  	KIIFLDPLKIILYLRYLPVLLTVYYAAITFGSLYVLNVSIENSFGKEPYNFDTLIVGLLY
          	:  .::** ::  **: *: ::*  **::**:*:::*::*: .:   **.:. : :**:*

MG09502.1 	IPSSLGYVISSIFGGRWIDYIMAREARKAERYDADGKLIYLPEDRMCENAWLAAALYPSA
NCU01411.1	LPSSLGYIVASLFGGRWTDKIMMREARNAGRYDADGNPIFLPEDRMRENIWLAASMYPAA
FG10120.1 	IPGGLGYIVGSLSGGRWIDKIMAREARKAGRYDEDGKLIYLPEDRMRENAWVMTTMYPLA
AN5656.1  	IPNSLGYVVSSIFGGRWIDKIMVREAKKANRYDEKGNLIFRPEDRMRENAWLGALLYPAG
          	:*..***::.*: **** * ** ***::* *** .*: *: ***** ** *: : :** .

MG09502.1 	LIMFGWTIDKGLHWAVP~SVASFLFGLSSMLIF~SAATTMLTEFMPSKSSSGIAVNNFVR
NCU01411.1	MVWFGWTAQHGVYWIVP~CVANFFFGCGSMLVF~GAVTTMLTEFMPHRSSSGVAVNNFVR
FG10120.1 	LLMFGWVLRYGLHPAVP~CVALFLFGISSMLVF0SVATTMLTELIRKRSSSGVAVNNFVR
AN5656.1  	LIWYGWTVDKGVFWLAP0MIANFFFGIGSMLIF~SMVTTMLTEFMPKKSSEGVALNNFTR
          	:: :**.   *:.  .*  :* *:** .***:* . .******::  :**.*:*:***.*

MG09502.1 	NIFSCVGTIVTQPLIDAMGIG~WLCTMIGLLALILGLGSVYSLKKFGPKWRKEMDRKLAI
NCU01411.1	NIFSCVGTIVAQPLINAMGIG~WLCTMIGLFAWVTGNLAIWLLKRNAAKWRESMDNALNT
FG10120.1 	NTLSCTGAIVAAPWINAIGTG2YIC--------------IFLLRKNATKWRKAMDKALGQ
AN5656.1  	NIFSCVGSFVTAPIIGAIGNG~WLFTIIGLVAFAS-SGVIVVMRVFGPRWKEGMDRLMQ-
          	* :**.*::*: * *.*:* * :: :  .  :       :  ::  ..:*:: **. :  

MG09502.1 	--
NCU01411.1	KT
FG10120.1 	--
AN5656.1  	--
          	
```
